# Bone marrow mesenchymal stem cells transfer in patients with ST-segment elevation myocardial infarction: single-blind, multicenter, randomized controlled trial

**DOI:** 10.1186/s13287-020-02096-6

**Published:** 2021-01-07

**Authors:** Runfeng Zhang, Jiang Yu, Ningkun Zhang, Wensong Li, Jisheng Wang, Guocai Cai, Yu Chen, Yong Yang, Zhenhong Liu

**Affiliations:** 1 Department of Cardiology, Department of Clinical Pharmacy, The Third Hospital of Mianyang/Sichuan Mental Health Center, Mianyang, 621000 Sichuan China; 2 Department of Cardiology, The Affiliated Hospital of Southwest Medical University, Luzhou, 646000 Sichuan China; 3 Heart Centre, The Navy General Hospital, Beijing, 100048 China; 4 Department of Cardiology, The General Hospital of Chinese People’s Armed Police Forces, Beijing, 100039 China

**Keywords:** Mesenchymal stem cells, Bone marrow, Stem cells transplantation, Myocardial infarction

## Abstract

**Objective:**

Our aim was to evaluate the efficacy and safety of intracoronary autologous bone marrow mesenchymal stem cell (BM-MSC) transplantation in patients with ST-segment elevation myocardial infarction (STEMI).

**Methods:**

In this randomized, single-blind, controlled trial, patients with STEMI (aged 39–76 years) were enrolled at 6 centers in Beijing (The People’s Liberation Army Navy General Hospital, Beijing Armed Police General Hospital, Chinese People’s Liberation Army General Hospital, Beijing Huaxin Hospital, Beijing Tongren Hospital, Beijing Chaoyang Hospital West Hospital). All patients underwent optimum medical treatment and percutaneous coronary intervention and were randomly assigned in a 1:1 ratio to BM-MSC group or control group. The primary endpoint was the change of myocardial viability at the 6th month’s follow-up and left ventricular (LV) function at the 12th month’s follow-up. The secondary endpoints were the incidence of cardiovascular event, total mortality, and adverse event during the 12 months’ follow-up. The myocardial viability assessed by single-photon emission computed tomography (SPECT). The left ventricular ejection fraction (LVEF) was used to assess LV function. All patients underwent dynamic ECG and laboratory evaluations. This trial is registered with ClinicalTrails.gov, number NCT04421274.

**Results:**

Between March 2008 and July 2010, 43 patients who had underwent optimum medical treatment and successful percutaneous coronary intervention were randomly assigned to BM-MSC group (*n* = 21) or control group (*n* = 22) and followed-up for 12 months. At the 6th month’s follow-up, there was no significant improvement in myocardial activity in the BM-MSC group before and after transplantation. Meanwhile, there was no statistically significant difference between the two groups in the change of myocardial perfusion defect index (*p* = 0.37) and myocardial metabolic defect index (*p* = 0.90). The LVEF increased from baseline to 12 months in the BM-MSC group and control group (mean baseline-adjusted BM-MSC treatment differences in LVEF 4.8% (SD 9.0) and mean baseline-adjusted control group treatment differences in LVEF 5.8% (SD 6.04)). However, there was no statistically significant difference between the two groups in the change of the LVEF (*p* = 0.23). We noticed that during the 12 months’ follow-up, except for one death and one coronary microvascular embolism in the BM-MSC group, no other events occurred and alanine transaminase (ALT) and C-reactive protein (CRP) in BM-MSC group were significantly lower than that in the control group.

**Conclusions:**

The present study may have many methodological limitations, and within those limitations, we did not identify that intracoronary transfer of autologous BM-MSCs could largely promote the recovery of LV function and myocardial viability after acute myocardial infarction.

## Introduction

Acute myocardial infarction (AMI) is a disease in which the coronary arteries suddenly interrupt the blood flow to the heart, causing acute and persistent ischemia and hypoxia in the heart, thus increasing the risk of death [1]. AMI is further divided into two subcategories: ST-segment elevation myocardial infarction (STEMI) and non-ST-segment elevation myocardial infarction (NSTEMI). STEMI is characterized by persistent typical ischemic chest pain and elevated serum myocardial necrosis markers, as well as typical ST-segment elevation of ECG [2–4], approximately accounting for 25–40% of AMI. Although most patients with STEMI can undergo percutaneous coronary intervention (PCI) and take the drugs recommended by the guidelines to relieve their symptoms, it cannot rescue the apoptotic and necrotic cardiomyocytes [5]. According to statistics, the risk of in-hospital death of AMI patients has not decreased significantly in the past 10 years [6], and the number of AMI patients in China will increase to 23 million by 2030 [7].

Increasing evidence indicates that stem cells have the ability of multi-directional differentiation, and it is getting more and more attention that stem cell transplantation serves as a new alternative therapy in repairing damaged myocardium [8]. For example, pluripotent stem cells, adult tissue stem/progenitor cells including endothelial progenitor cells, skeletal muscle myoblasts, cardiac stem/progenitor cells, and bone marrow mononuclear cells have been reported to participate in the treatment of damaged cardiomyocytes [9]. In addition, it was found that these cells can repair damaged myocardium through paracrine [10].

Bone marrow mesenchymal stem cells (BM-MSCs) are the most widely studied MSC types. Many experimental studies have shown that BM-MSCs can improve heart function after AMI [11–13]. Although BM-MSCs are not abundant in bone marrow nucleated cells, accounting for only 0.01%, they can be expanded one billion times in vitro without losing stem cell activity [14]. It had been found that BM-MSCs not only differentiated into cardiomyocytes to promote cardiomyocyte regeneration when they were transplanted into the heart in vivo [15, 16], but also secreted growth factors, cytokines, chemokines, and microRNAs to improve tissue microenvironment and effectively reduce the adverse remodeling and inflammation of cardiomyocytes. Additionally, the secreted proteins of BM-MSCs have immunosuppressive properties by regulating T cells, B cells, and monocytes [17–20]. Clinically, autologous bone marrow MSCs have been adopted to treat myocardial infarction by transplanting after the expansion [7]. However, reviewed from the clinical study of BM-MSCs on AMI from 2004 to 2017, it was found that only two clinical research results showed that BM-MSCs can increase the left ventricular ejection fraction of patients compared with the control group [21]. Based on existing experimental and clinical data, the best time for transplantation was 7 to 14 days after acute myocardial infarction [22–24], and it took at least 14 days to cultivate the BM-MSC seed cells. Therefore, we select to perform BM-MSC transplantation within 1 month after PCI. We chose to use BM-MSC injection with independent intellectual property rights in China to conduct a randomized, single-blind, parallel-controlled multicenter clinical trial to observe the effectiveness and security of BM-MSC transplantation in the treatment of acute myocardial infarction. It is hoped that this study will show whether BM-MSC injection transplantation is effective and safe in patients with STEMI and provide a reliable basis for clinical promotion.

## Methods

### Participants

**Fig. 1:**
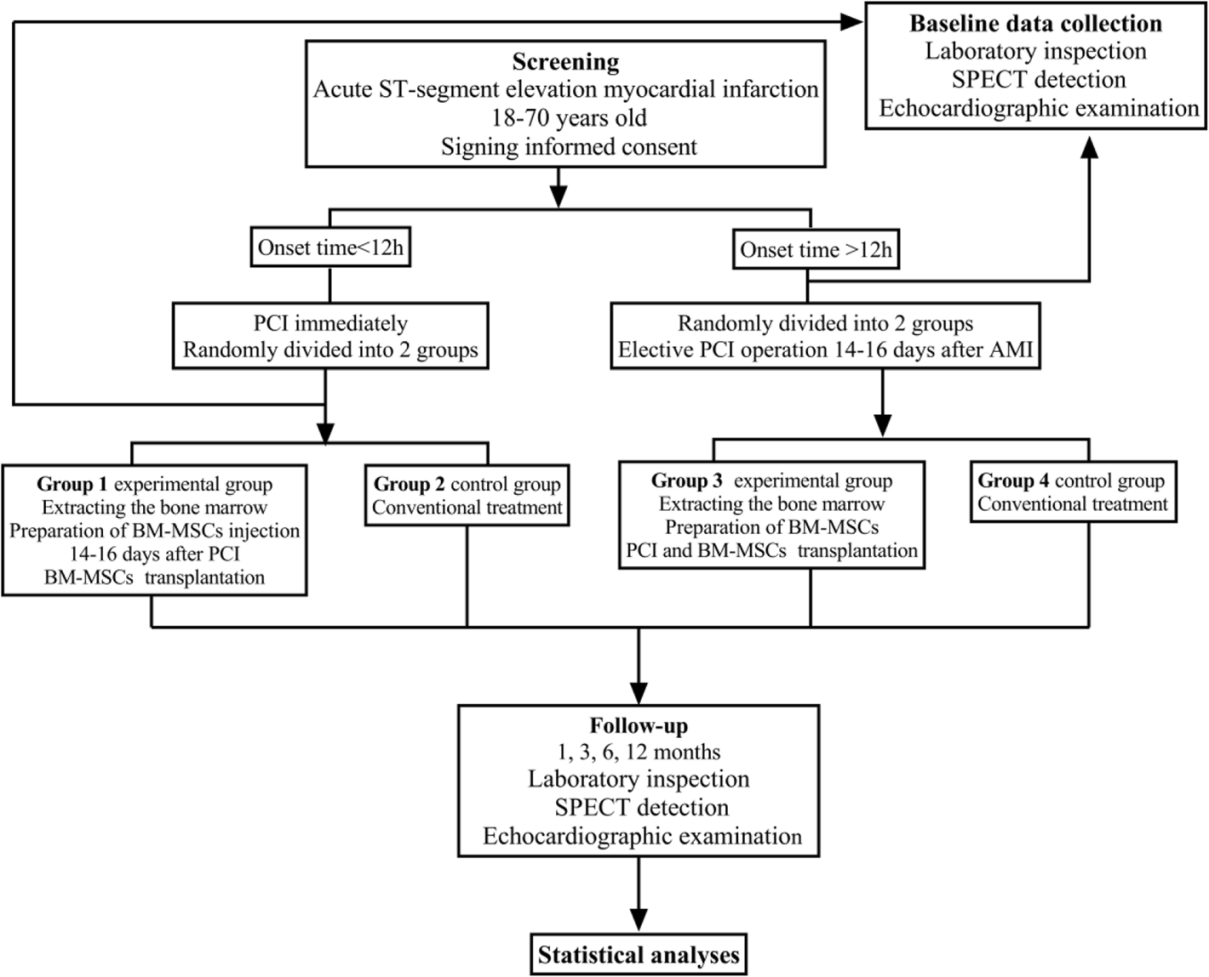
Research protocol

This study is a randomized single-blind, parallel-controlled multicenter clinical trial that started in March 2008 and ended in July 2010. A total of 43 patients came from 6 hospitals in Beijing (The People’s Liberation Army Navy General Hospital, Beijing Armed Police General Hospital, Chinese People’s Liberation Army General Hospital, Beijing Huaxin Hospital, Beijing Tongren Hospital, Beijing Chaoyang Hospital West Hospital), including 39 males and 4 females. Their age ranged from 39 to 76 years, with an average age of 58.9 ± 11 years. Based on the agreement of the Center Clinical Trial Ethics Committee and the Helsinki Declaration, we came to the research protocol as shown in Fig. 1. The standards of getting enrolled are STEMI patients, older than 18 years, who had an onset time of less than 1 month and was successfully revascularized with infarct-related vascular blood flow returning to TIMI level 3. All patients enrolled in the study signed an informed consent form and promised to complete all follow-up plans. Exclusion criteria include the following eight items: (1) patients with refractory persistent ventricular tachycardia; (2) patients with high heart block and not controlled by pacemaker; (3) patients with hepatic or kidney dysfunction (ALT > 80 U/L, creatinine (Cr) > 440 mmol/L); (4) patients with bleeding disorders or malignant tumors; (5) patients with autoimmune diseases or any serious fatal disease; (6) patients with contraindications for coronary intervention; (7) patients with the following other heart diseases: congenital heart disease (such as ventricular defect, atrial defect, patent ductus arteriosus), primary heart valve disease, active myocarditis, pulmonary heart disease, hyperthyroid heart disease, mucoedema heart disease, etc.; and (8) patients with mental illness, no self-awareness, and no precise expression and cooperation.

### Randomization and study treatment

Participants’ random numbers were generated by the network, and technical services were provided by the China Cardiovascular and Cerebrovascular Diseases Professional Network (CCVD), which was not related to this clinical trial. The participant’s information was input into the computer. If the patient met the inclusion criteria, the system would give a random number and grouping to determine the randomization of the patient. The 43 patients were randomly divided into a cell transplantation group (BM-MSC injection via coronary artery perfusion, *n* = 21) and a control group (all other treatments except cell transplantation were the same as the cell transplantation group, *n* = 22). Because of ethical considerations, we decided not to conduct bone marrow aspiration and left heart catheterization in patients randomized to the control group. Those who were unsuccessful in revascularization were withdrawn from this study.

### Preparation of BM-MSC injection

The collection and separation of bone marrow are performed in a sterile room. Under lidocaine local anesthesia, 80 ml of bone marrow was extracted from the patient’s posterior superior iliac crest and placed in 2000 IU heparin saline. The BM-MSC injection was prepared by the Stem Cell and Regenerative Medicine Center of the Institute of Field Transfusion of the Academy of Military Medical Sciences according to standard procedures. Firstly, the extracted bone marrow was subjected to natural sedimentation, low-temperature centrifugation, saline washing, resuspension, counting, and Percoll centrifugation to obtain mononuclear cells. Then, mononuclear cells were cultured in DMEM medium with 10% fetal bovine serum to obtain BM-MSCs, and they were subcultured when the stem cells grew to 80% confluence. Next, take the cells after the 2–3 subculture and expansion for 72 h, and at the same time, take the culture supernatant for bacteria, mold, and mycoplasma identification tests. The next step was to digest the cells with 0.25% trypsin at 37 °C. After repeated washing with normal saline for 3 times, we then calculated the cell concentration, the ratio of living cells, and verified the cell phenotype. Finally, resuspend the cells with 2 ml of normal saline for injection, place them in a 2-ml vial, and mark the patient’s name and product number to ready for transplantation. The specification of this product was 1.0~2.5 × 10^6^ BM-MSCs/2 ml, 2.0 ml/bottle. During the operation, 4 ml of BM-MSC injection was diluted to 10 ml.

### Injection of BM-MSCs via coronary artery

The preoperative preparation of BM-MSCs undergoing coronary artery transplantation is the same as PCI. 14.07 ± 9.53 days after PCI, firstly, the patient was inserted with an ultra-long guide wire and inserted into the guide wire balloon catheter (OTW balloon) along the extra-long guide wire to the distal end of the stent. Then, pull out the guide wire and inflate the balloon pressure until there is no forward blood flow in the target vessel (balloon inflation period). Finally, to facilitate the transplantation of cells through the endothelial channel and migration into the infarcted zone, when the target vessel was completely occluded, 2 ml of BM-MSC suspension was infused by high-pressure injection directly into the necrotic area along the central lumen of the guidewire balloon catheter. During the operation, the balloon was kept inflated for 2 min at a time to block the blood flow; the transplanted cells were not washed away immediately under these conditions, and then the perfusion was restored for 2 min to reduce the likelihood of ischemia to a minimum [23]. The above process was repeated 6 to 8 times, and the patient did not undergo angiography again after the stem cell implantation. Patients were monitored for chest pain, changes in ECG, and intracavitary pressure changes during surgical procedures.

### Echocardiographic examination

The subjects underwent echocardiographic examinations before and 12 months after the operation, and the left ventricular end-diastolic volume, end-systolic volume, and ejection fraction was measured by the Simpson method. The color heart ultrasound system (GE, USA) uses VIVID7, the probe is S4, and the frequency is 2~4 MHz. We would collect four standard two-dimensional images (the parasternal long axis and short axis, apical two-chamber and four-chamber view).

### Myocardial perfusion-metabolic imaging examination

The purpose of cardiac perfusion-metabolic imaging was to evaluate the nature of left ventricular myocardial perfusion-metabolic defects (reversible defects, fixed defects) and changes in area before and after BM-MSC transplantation. The SPECT image was divided into 20 segments, and the score was determined according to the degree of attenuation of myocardial nuclide uptake in each segment (0 points = adequate intake, 1 point = slight decrease in intake, 2 points = moderate reduction in intake, 3 points = reduced ingestion, 4 points = intake defect). The sum of the scores of each segment was obtained to quantify the evaluation index of myocardial perfusion-metabolic defect, that is, the higher the value, the larger the range of myocardial perfusion-metabolic defect. The cardiac metabolic imaging was read by a professional nuclear medicine physician.

Patients with hyperlipidemia were treated with oral hypolipidemic drugs (inositol niacinate 0.2 g or reserpine) 2 h before the examination. If the patient had diabetes, control the food he took to a low level 1 h before the examination. We used the automatic blood glucose meter to measure the blood sugar. According to the patient’s blood sugar situation, oral glucose or subcutaneous injection of insulin was considered for blood glucose regulation, and finally the patients’ blood glucose was controlled in the range of 7.8 to 8.8 mmol/L. The participants were intravenously injected with the imaging agent ^99^mTc-MIBI 20Mci (740 MBq) and ^18^F-FDG 8~10 Mci (296–370 MBq) after 30 min of blood glucose regulation. Myocardial perfusion-metabolic imaging was observed after 45–60 min of injection. The ^99^mTc-MIBI (^99^mTc-methoxyisobutyl isonitrile) and ^18^F-FDG (^18^F-deoxyglucose) was provided by Atomic High Tech of China Institute of Atomic Energy. The imaging instrument used GE’s Millennium VG Hawkeye SPECT, equipped with 511 Kev high-energy collimator and dual probes in L mode. The acquired images were processed by the ECToobox heart software, and the horizontal long-axis, vertical long-axis, short-axis images, and bullseye image were obtained after reconstruction.

### Observation

#### Primary endpoint

The changes of myocardial metabolic activity (SPECT detection) at the 6th month after autologous BM-MSC transplantation and the changes in left ventricular ejection fraction (LVEF) at the 12th month after transplantation of autologous BM-MSCs.

#### Secondary endpoint

Incidence of cardiovascular events, overall mortality, and adverse events 12 months after transplantation of autologous BM-MSCs.

### Evaluation index

#### Effective assessment of myocardial reconstruction: assessment using cardiac metabolic imaging

The following two conditions are indispensable before BM-MSC transplantation in myocardial reconstruction can be judged to be effective: (1) the increase in 18-FDG uptake at the 6th month after BM-MSC transplantation constitutes a statistical difference with that before transplantation and (2) the increase in 18-FDG uptake at the 6th month after BM-MSC transplantation constitutes a statistical difference with the control group.

#### Effective evaluation of improving heart function: assessment using LVEF

The following two conditions are indispensable before BM-MSC transplantation in improving cardiac function can be judged to be effective: (1) the increase in LVEF at the 12th month after BM-MSC transplantation constitutes a statistical difference with that before transplantation and (2) the increase in LVEF at the 12th month after BM-MSC transplantation constitutes a statistical difference with the control group.

The safety was evaluated by coronary angiography, laboratory abnormalities, and the incidence of adverse events during the 12 months’ follow-up.

### Statistical analyses

Statistical analysis was performed with SPSS17.0 software. The measurement data were expressed as mean ± standard deviation (mean ± SD), and the comparison of means between the two groups was analyzed by *t* test; *P* < 0.05 was considered a significant difference.

## Result

### Research protocol

#### Patient characteristics

**Fig. 2:**
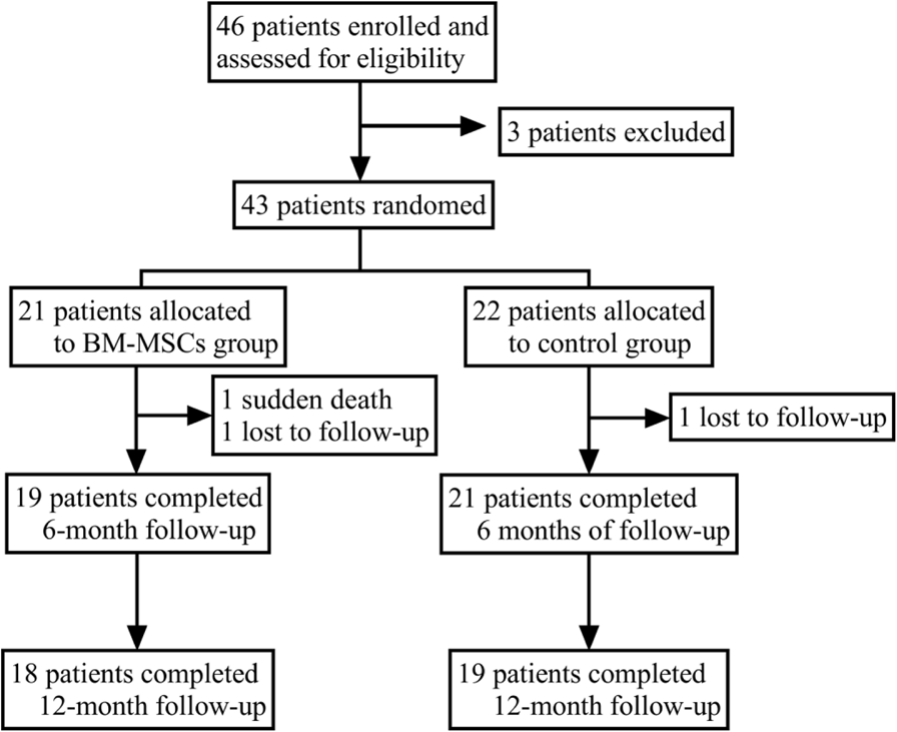
Enrollment and outcomes

The enrollment and outcomes of the patients are shown in Fig. 2. In the BM-MSC transplantation group, 19 patients completed a 6-month follow-up and 18 patients completed a 1-year follow-up. In the control group, 21 patients completed a 6-month follow-up, and 19 patients completed a 1-year follow-up. One case in each group did not involve in the 6-month follow-up for the patients’ emigration. One patient died in the BM-MSC transplantation group. For patients with acute myocardial infarction < 12 h, baseline data were collected after direct PCI, before BM-MSC transplantation, and the patient’s condition was stable. For onset > 12 h, the baseline data were collected before PCI. There were no significant differences between the baseline clinical characteristics of the two groups of subjects (Table 1).

**Table 1:**
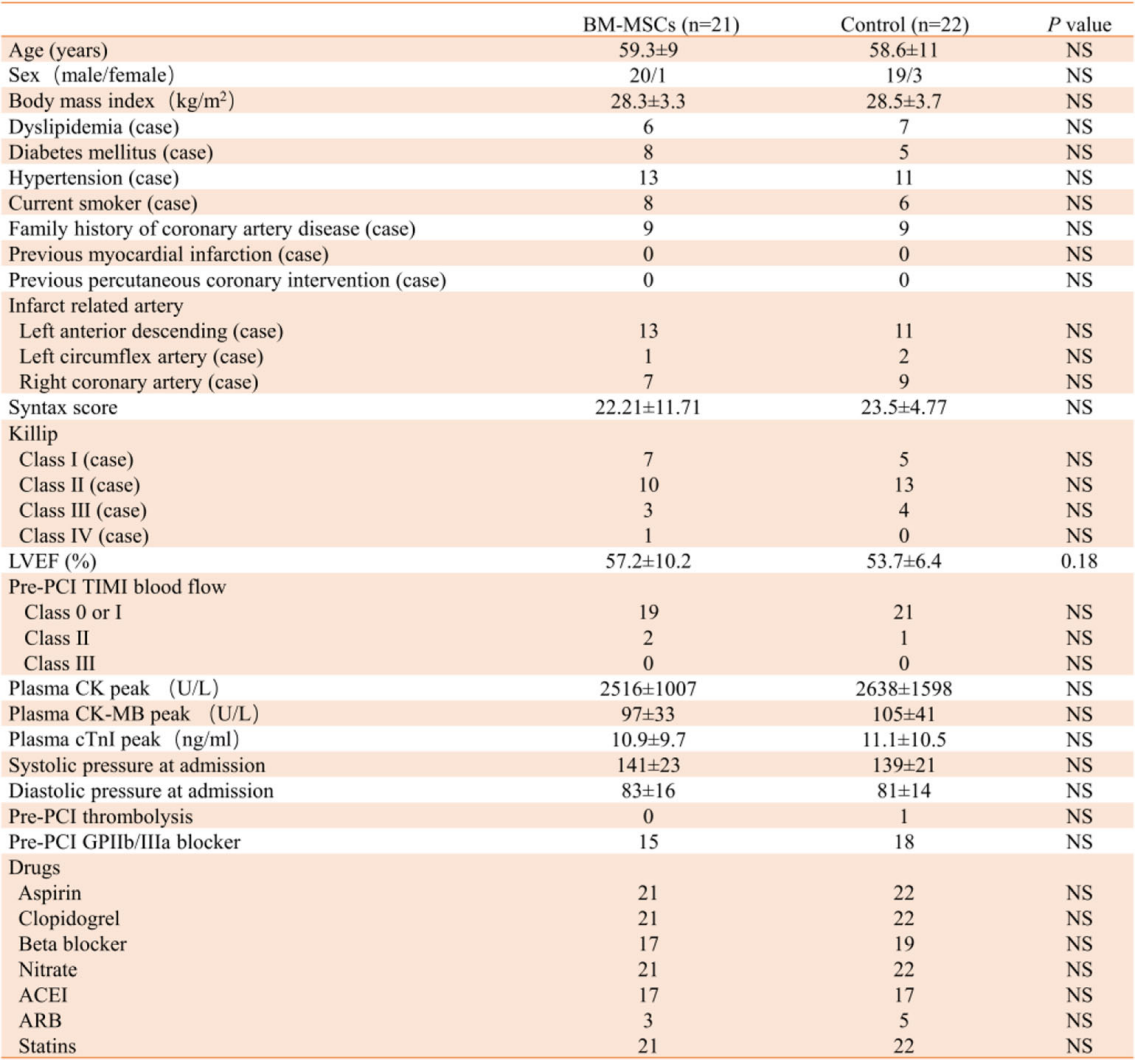
Baseline characteristics of the study population

### Comparison of echocardiographic parameters before and after surgery in two groups

**Fig. 3:**
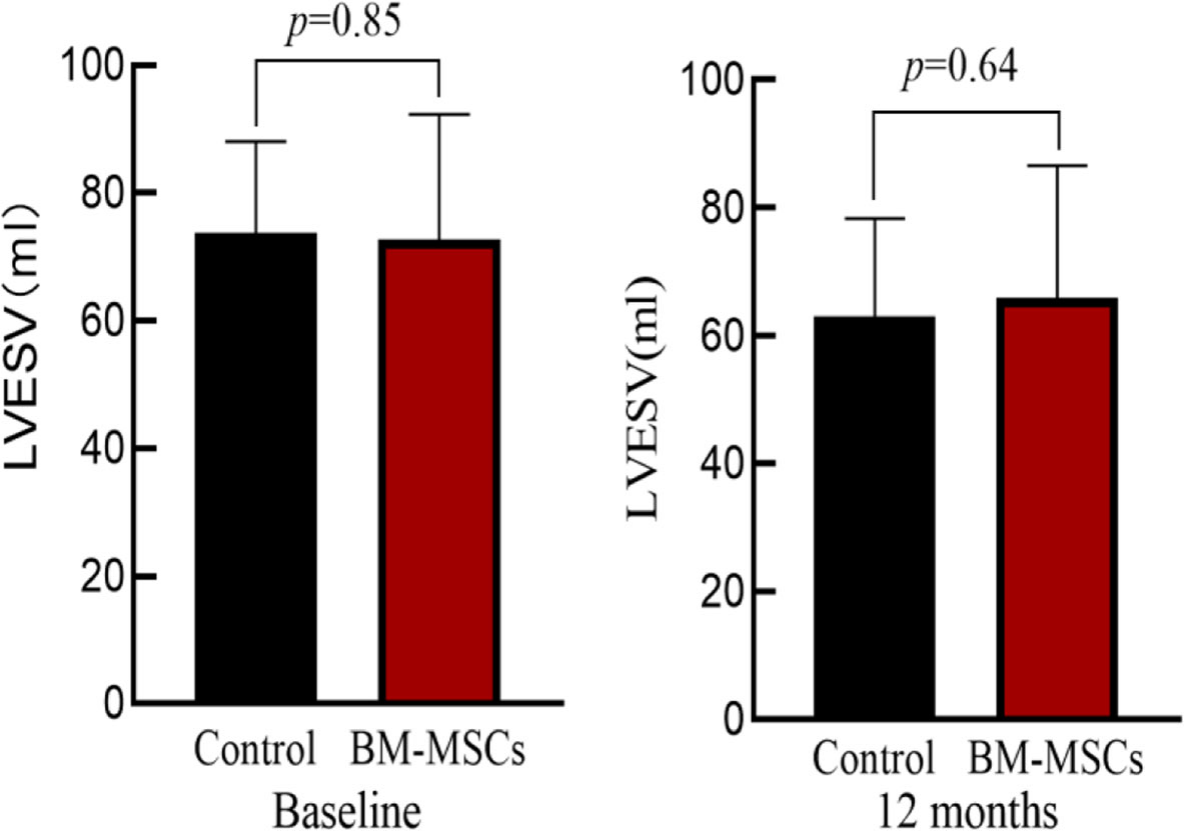
LVESV comparison

**Fig. 4:**
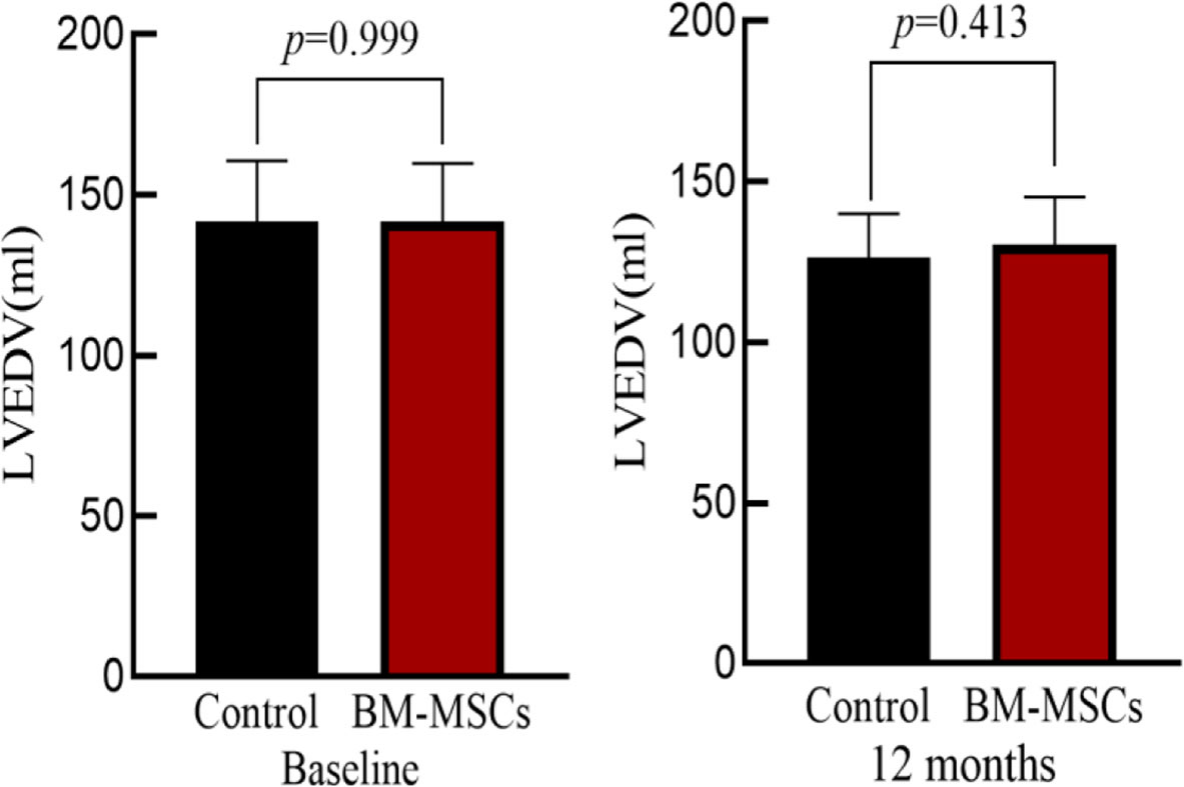
LVEDV comparison

**Fig. 5:**
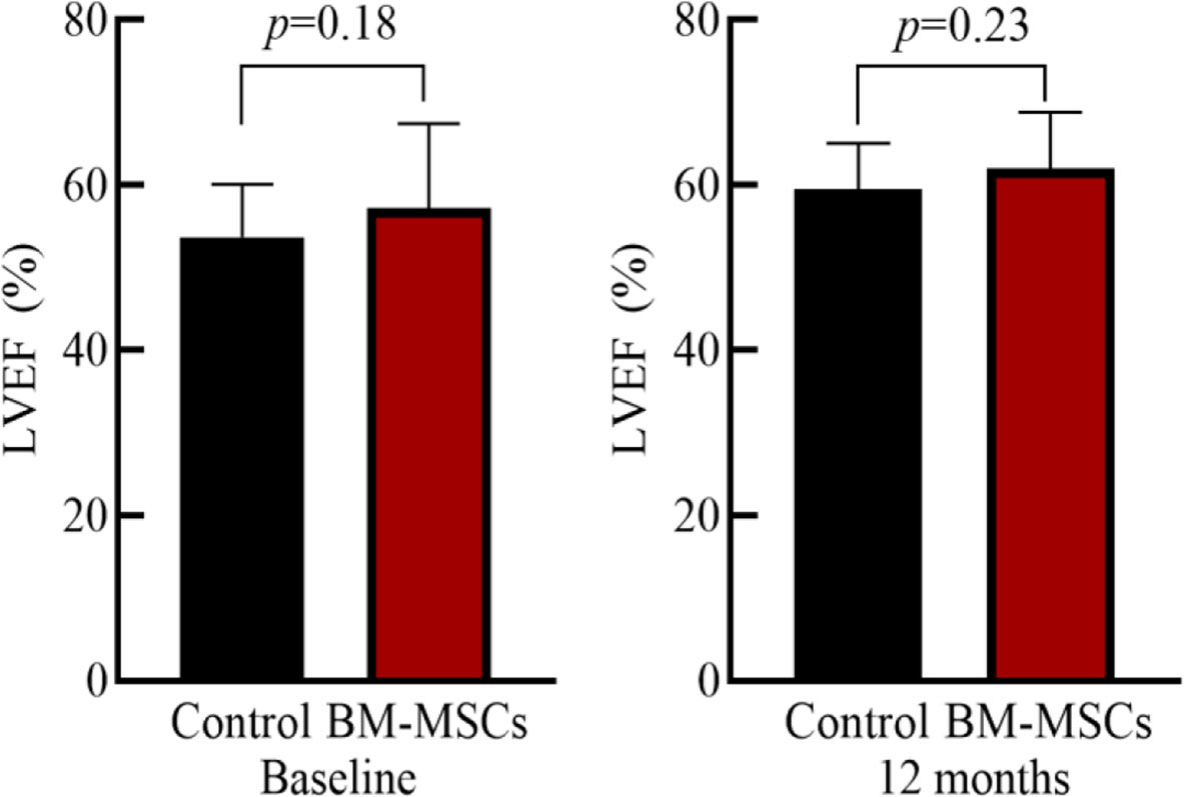
LVEF comparison

There was no significant difference in left ventricular end-systolic volume (LVESV), left ventricular end-diastolic volume (LVEDV), and LVEF between the two groups at baseline (*P >* 0.05). After 12 months, the LVESV, LVEDV and LVEF in the BM-MSCs group was significantly improved compared with that before transplantation. The control group also showed the same results as the BM-MSC group, but there was no significant differences in LVESV, LVEDV, and LVEF improvement in two groups (*P >* 0.05) (Table 2, Figs. 3, 4, and 5).

**Table 2:**
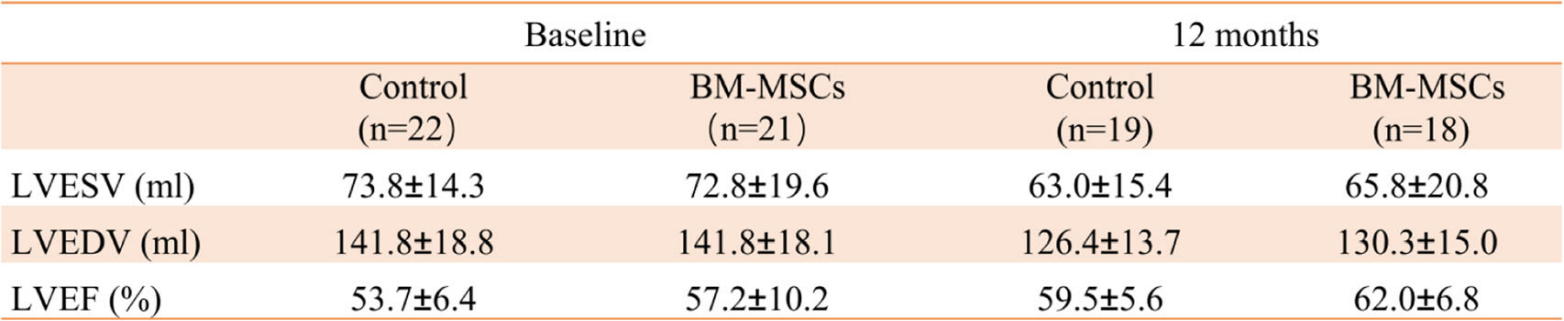
Comparison of cardiac function between BM-MSC group and control group

### Comparison of myocardial perfusion and metabolic activity between the two groups

**Fig. 6:**
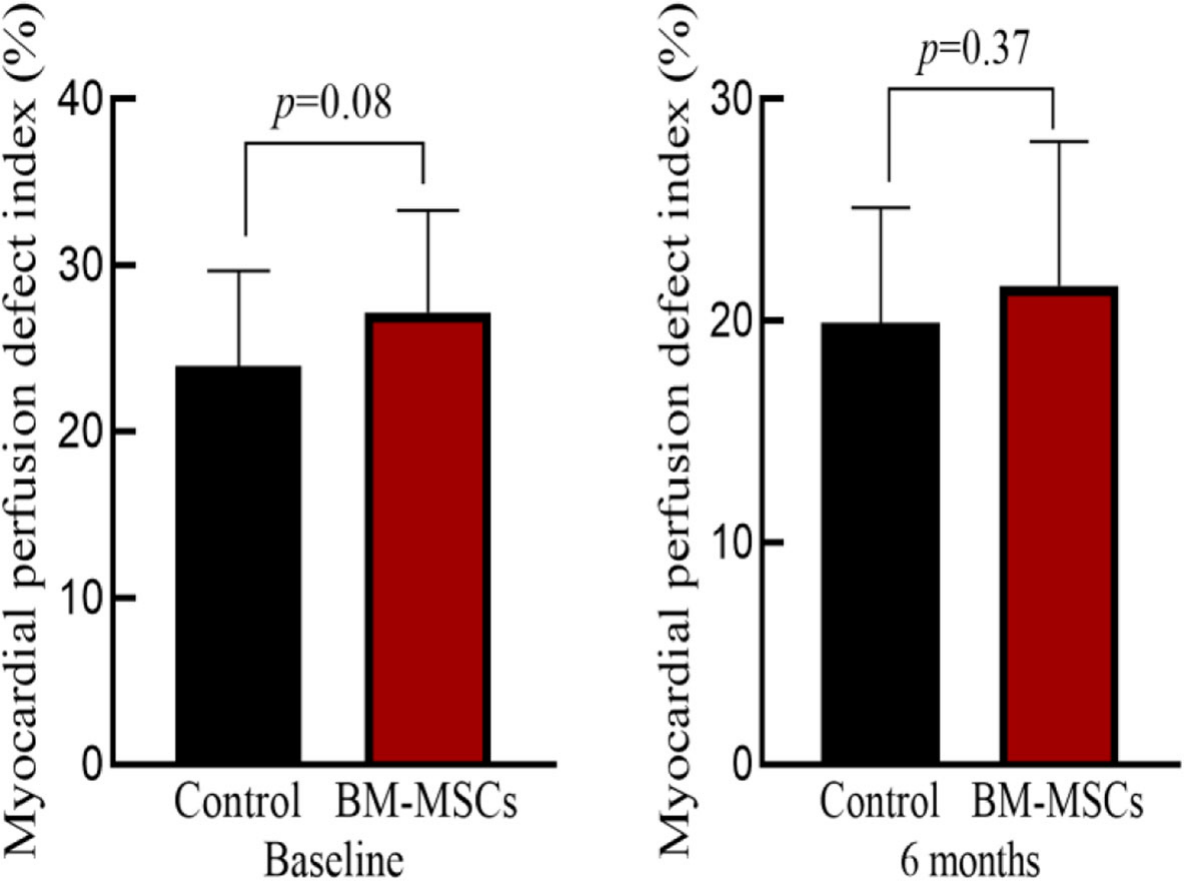
Myocardial perfusion defect index

**Fig. 7:**
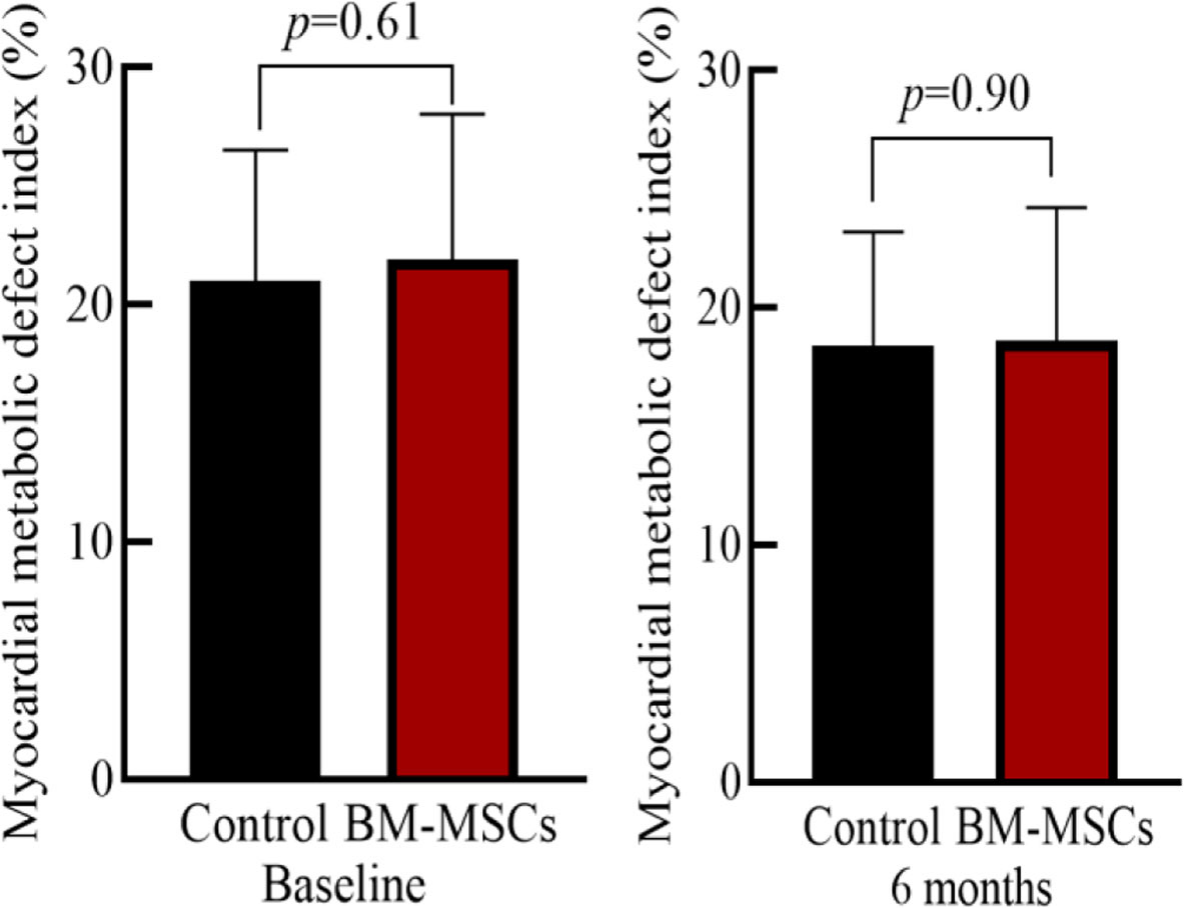
Myocardial metabolic defect index

**Fig. 8:**
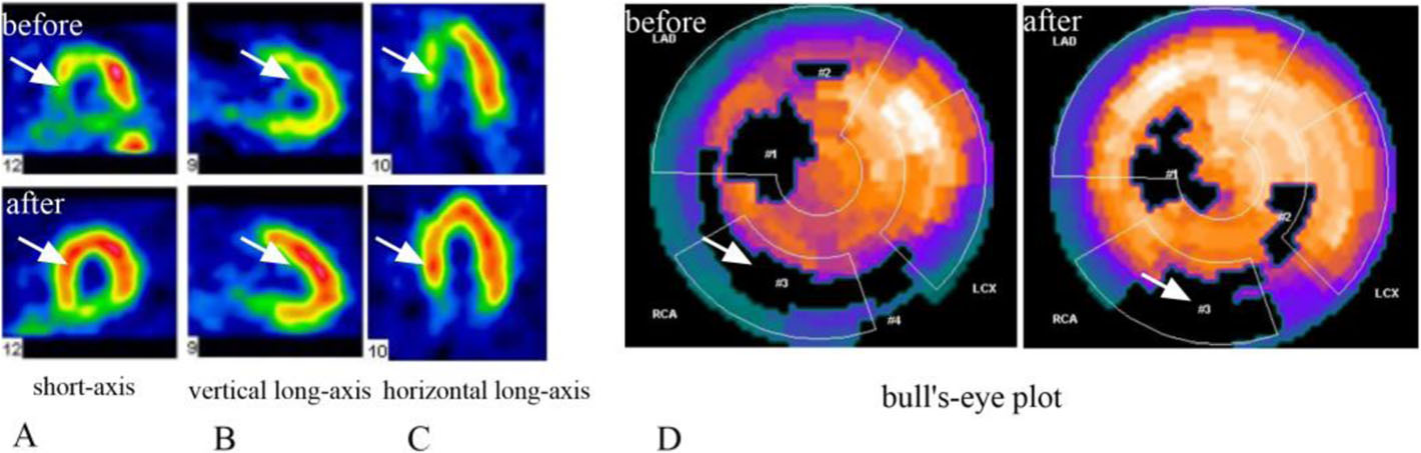
Representative ^99^mTc-MIBI positron emission tomograms in **a** short-axis view, **b** vertical long-axis view, **c** horizontal long-axis view, and **d** bull’s-eye view. Before operation, the multiple areas showed persistent defects (arrows). Six months after cell transplantation, the same area show a restoration of ^99^mTc-MIBI uptake, suggesting a return of the myocardial blood perfusion with increased tracer intensity in the infarcted area after versus before cell infusion

**Fig. 9:**
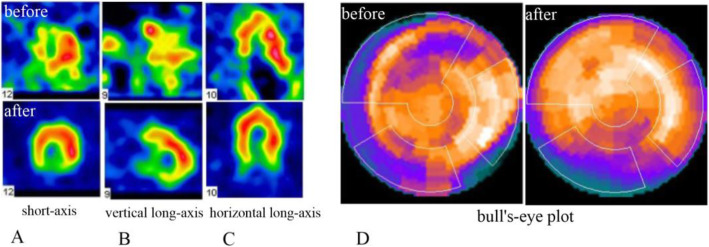
Representative ^18^F-FDG positron emission tomograms in **a** short-axis view, **b** vertical long-axis view, **c** horizontal long-axis view, and **d** bull’s-eye view. Before operation, the multiple area showed a sustained deficit. Six months after cell transplantation, the same areas show a restoration of glucose uptake, suggesting a return of myocardial viability with increased tracer intensity in the infarcted area after versus before cell infusion

The myocardial perfusion and metabolic defect index at baseline of the two groups of patients was similar (*P* > 0.05). There was no significant difference between the two groups before and after treatment (*P* > 0.05). Compared with the control group, the myocardial perfusion and metabolic defect in the BM-MSC transplantation group did not improve after 6 months (*P* > 0.05), suggesting that the infarcted myocardium was not effectively replaced or repaired (Table 3, Figs. 6, 7, 8, and 9).

**Table 3:**
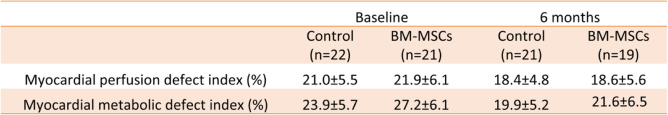
Comparison of static myocardial perfusion-metabolic defect index before and after treatment in BM-MSC transplantation group and control group

### Follow-up results and safety assessment

During the follow-up period, the types and numbers of arrhythmias detected by Holter were similar in the two groups (Table 4). In the BM-MSC group, there was no significant difference in blood leukocyte (WBC), Cr, and carcinoembryonic antigen before and after transplantation, and ALT and CRP were significantly decreased (Table 5). There was no statistical difference between the BM-MSC group and the control group in the above laboratory examination indexes after the end of the experiment (Table 6).

**Table 4:**
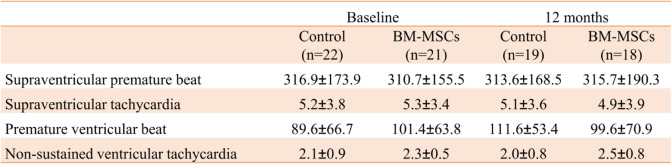
Comparison of Holter between BM-MSC group and control group

**Table 5:**
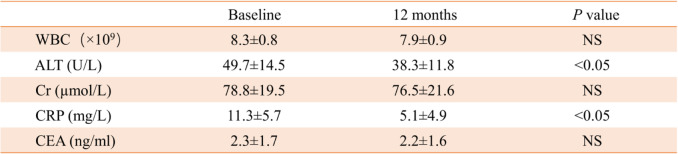
Changes of laboratory indexes before and after transplantation in BM-MSC group (*n* = 18)

**Table 6:**
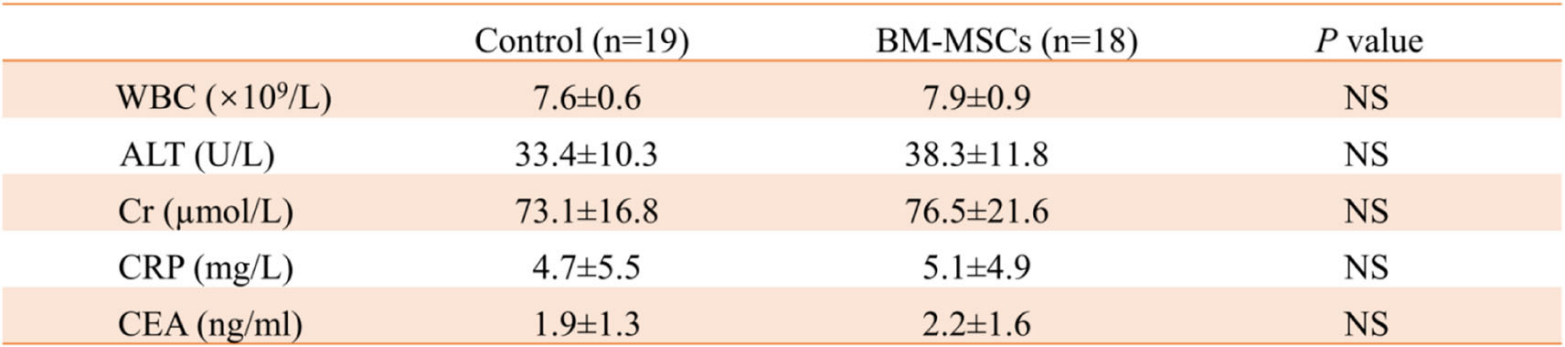
Comparison of laboratory indexes between BM-MSC group and control group

There were no adverse reactions such as stent thrombosis, recurrence of myocardial infarction, malignant arrhythmia, tumor, and myocardial fibrosis in the two groups of patients during the peri-treatment period. One case of sudden death in the BM-MSC group and one patient occurred microvascular embolism (Table 7).

**Table 7:**
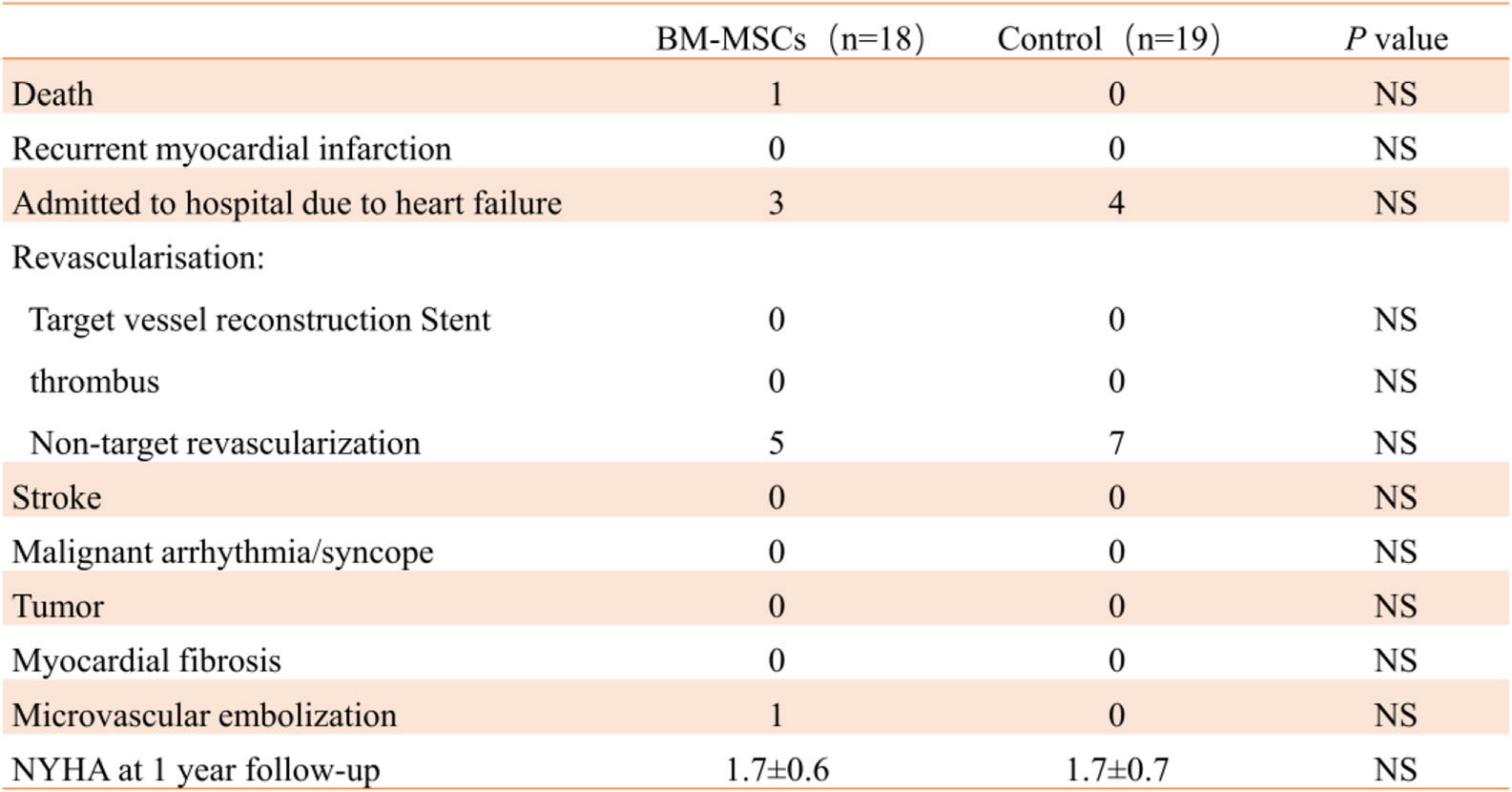
Cumulative clinical events during the 12-month follow-up period in both groups

## Discussion

In the field of clinical research on stem cells, there are different reports on the types of transplanted cells, and there are also differences in the results of BM-MSCs cell transplantation on the improvement of cardiac function in clinical studies. In this study, autologous BM-MSCs (prepared uniformly by the Stem Cell and Regenerative Medicine Center of the Field Transfusion Institute of the Academy of Military Medical Sciences) were selected and transplanted into the heart after infarction by intracoronary injection. The effect and safety of the transplantation were observed. The results showed that compared with the control group, the 6-month metabolic imaging defect score of the BM-MSC transplantation patients was not statistically significant, and the 12-month left ventricular ejection fraction did not constitute a statistically significant improvement. The reasons for the above results are as follows:

1. It is related to the insufficient number of the enrolled patients. The number of selected cases was determined on the basis that the differences between the different treatment groups when the primary endpoint which is expected to reach is 4 to 5%. If the difference between the different treatment groups was 4.5%, the bilateral test was carried out at a significant level of *α* = 0.05, and the test efficacy reached 80%. To draw a conclusion, the difference was statistically significant; then, 34 patients were required in each group [25]. Our initial trial design plan was to distribute the group serial numbers to 6 research centers to ensure that there are at least 240 clinically valid cases (120 pairs) at the end of the experiment. Each trial center has completed at least 40 clinically valid cases (20 pairs). During the implementation of this study, it was discovered that although the researchers explained in detail to the patients that the extraction of bone marrow had no adverse effects on the body, the patient had an instinctive fear of taking bone marrow, and they were very resistant. In addition, since this study was an innovative clinical trial, the postoperative efficacy was unknown. In this study, therefore, the number of patients we recruited was limited—only 43 patients had been eventually enrolled in 6 hospitals over the past 2 years, and the number of cases was seriously insufficient.
2. It is related to insufficient dose of transplanted cells. The number and function of autologous bone marrow MSCs decrease with age [26]; 80 ml bone marrow was extracted from the patient, extracted, cultured, and expanded for 2 weeks (13.89 ± 1.60 days) to obtain 1~2.5 × 10^6^ BM-MSCs. At the same time, considering that high-dose cell transplantation may cause embolization of distal blood vessels [27], we chose low-dose cell transplantation (3.31 ± 1.70 × 10^6^ cells). The number of cells may not reach the therapeutic dose. Perin et al. [28] compared the effects of three doses (25, 75, or 150 × 10^6^ cells) of allogeneic BM-MSCs on adverse cardiac events and left ventricular remodeling. The results showed that the higher the dose of BM-MSCs, the fewer adverse cardiac events, the lesser the degree of left ventricular remodeling. In addition, the TRIDENT trial found that compared with the lower dose of BM-MSCs (20 × 10^6^ cells), the higher dose group of BM-MSCs (100 × 10^6^ cells) can improve LVEF and maintain the level of serum brain natriuretic peptide [29]. It is worth noting that the abovementioned experiments showed that the number of cells with significantly improved cardiac function after BM-MSC transplantation was greater than 70 × 10^6^ cells. However, the number of cells transplanted into the patient in this experiment was 3.31 ± 1.70 × 10^6^ cells, which was much smaller than 70 × 10^6^ cells. Therefore, compared with the control group, the experimental group was more likely to have a negative result.
3. The optimal time window for cell transplantation may have been missed. The appropriate time for stem cell transplantation should be after the inflammatory response and before the scar extension. Premature transplantation may result in the death of a large number of transplanted cells due to the poor local microenvironment, which may affect the transplantation effect. If transplantation is too late, local damage may be irreversible and ventricular remodeling may have been formed [30]. Previous clinical research data suggest that the clinical research of intracoronary stem cell injection therapy should be conducted 7 to 14 days after acute myocardial infarction [22–24]. However, the culture of BM-MSCs in this experiment required at least 14 days, thus the stem cell transplantation time was 14.07 ± 9.53 days after PCI and 23.24 ± 7.69 days after myocardial infarction, which was beyond the operating time range for obtaining positive experimental results. In addition, some scholars believe that the best time for stem cell transplantation is 4~7 days after the onset time of STEMI [31]. The latest meta-analysis results also show that [32] the optimal time window for BM-MSC transplantation was within 1 week after PCI. However, Kim et al. [33] transplanted autologous BM-MSCs 30 ± 1.3 days after PCI and found that compared with the control group, the BM-MSC group can significantly increase the left ventricular ejection fraction of the patient. Therefore, further clinical randomized trials need to confirm the optimal transplant time window to treatment.
4. It may not be the optimal way for BM-MSC transplantation in our experiments. Fukushima et al. [34] and others found that the transplantation methods of BM-MSC, such as intramyocardial injection, intracoronary injection, or intravenous injection, are not satisfactory. There is still a need to develop new and more effective cell transplantation methods, such as the use of bioengineering technology for epicardial implantation [34–36]. In this study, BM-MSCs were transplanted into coronary arteries, and the number of cell survival was unknown [37], which affected the results of the experimental group.
5. It may not be the optimal seed cell. BM-MSC is a subgroup of bone marrow stem cells with low bone marrow content and needs to be cultured and expanded in vitro. In particular, BM-MSCs need to be cultured for a long time, which brings about in vitro pollution; reduced homing function, large cell size, easy microthrombosis, and other undesirable factors; and reduces which their better cardiac regeneration effect. Therefore, the use of autologous bone marrow mesenchymal stem cells as seed cells for cell transplantation in this study may have great limitations and potential risks, and it is not a suitable cell type for transplantation. Current research shows that umbilical cord Wharton’s jelly-derived mesenchymal stem cells (WJ-MSCs) are a group of adult stem cells that are more primitive and have stronger differentiation potential than BM-MSCs [38]. Compared with BM-MSCs, WJ-MSCs are easier to obtain in large quantities and are more primitive. They have higher separation efficiency and stronger plasticity. They are stronger in repairing myocardium, regenerating blood vessels, and improving heart function [39, 40]. At present, many researchers believe that WJ-MSCs are true adult primitive stem cells between embryonic stem cells and adult stem cells. Several animal experiments have confirmed that WJ-MSC transplantation reduces cell apoptosis and fibrosis, strengthens viable myocardium, and improves the remodeling and function of ventricle. For example, Zhang et al. [41] evaluated the impact of WJ-MSC in a pig model of acute myocardial infarction and followed-up for 6 weeks. Lopez et al. [40], in a rat model of acute myocardial infarction, suggested that intravenous injection of WJ-MSC is more beneficial than BM-MSC to improve myocardial ischemia. What is more noteworthy is that although WJ-MSCs are allogeneic seed cells, they have immunogenic characteristics different from other xenogeneic stem cells, special immunosuppressive, and immunoregulatory functions and have no immunogenicity for transplantation in vivo [42]. However, there is still a lack of clinical trials to ensure the safety and effectiveness of this seed cell.

There was no significant difference between the incidence of cardiovascular events, the total mortality, and incidence of adverse events in the BM-MSC group and the control group. One patient in the BM-MSC group had a small number of BM-MSCs, the culture time was extended to 19 days, and coronary microembolization occurred during coronary artery transplantation. The analysis may be due to the long culture time of BM-MSCs, which reduces the function and quality of cells, resulting in increased volume and enhanced adhesion, resulting in microthrombosis and microvascular spasm [43, 44]. One case of death occurred in the BM-MSC group. When the patient was admitted to the emergency department, coronary angiography showed that the culprit’s blood vessels (the left anterior descending coronary artery and the left circumflex coronary artery) had a stenosis of 90% and the syntax score was 33. Therefore, coronary artery bypass graft (CABG) therapy was recommended. However, after communicating with patients and their families, PCI was chosen. The patient felt tightness and moderate edema of both lower limbs after the PCI. We consider the onset of acute left heart failure. Two weeks after PCI, the patient’s condition was stable. With the consent of the patient and his family, the patient received coronary BM-MSC injection therapy. The patient had no symptoms of particular discomfort during or after the BM-MSC injection therapy. Before and after the BM-MSC injection therapy, the patient tried to defecate slowly and did not want to take laxative drugs. On the third day after the operation, the patient suddenly fainted to the ground after getting up from the toilet. After discussion, the possibility of sudden cardiac death due to acute increase in cardiac load after the patient got out of bed to defecate cannot be ruled out. Unfortunately, no autopsy was performed to prove it.

The results of this study indicate that low-dose BM-MSC intracoronary transplantation is not inferior to traditional standard treatment after PCI for left ventricular function and myocardial remodeling after myocardial infarction. Under the condition that the BM-MSC culture time length is appropriate, the incidence of cardiovascular events, total mortality, and incidence of adverse events in patients with ST-segment elevation myocardial infarction will not be increased.

Based on the existing reports and the results of this experiment, BM-MSC is still an important donor for AMI cell transplantation therapy. However, if BM-MSCs are to achieve better therapeutic effects in the future, it is still necessary to improve the treatment regimen to enhance the therapeutic effect, such as the use of the optimal cell dose, optimal cell transplantation method, optimal seed cell, and the improvement of cell culture protocols to expand the BM-MSCs do not lose cell function, which requires further experimental and clinical studies to determine.

## Conclusions

The present study may have many methodological limitations, and within those limitations, we did not identify that intracoronary transfer of autologous BM-MSCs could largely promote the recovery of LV function and myocardial viability after acute myocardial infarction.

## Data Availability

The datasets used and/or analyzed during the current study are available from the corresponding author on reasonable request.

## Abbreviations

BM-MSCs: Bone marrow mesenchymal stem cells
STEMI: ST-segment elevation myocardial infarction
NSTEMI: Non-ST-segment elevation myocardial infarction
LV: Left ventricular
SPECT: Single-photon emission computed tomography
SD: Standard difference
ALT: Alanine transaminase
CRP: C-reactive protein
AMI: Acute myocardial infarction
PCI: Percutaneous coronary intervention
CCVD: Cerebrovascular Diseases Professional Network
LVEF: Left ventricular ejection fraction
LVESV: Left ventricular end-systolic volume
LVEDV: Left ventricular end-diastolic volume
CABG: Coronary artery bypass graft
